# Efficacy and Efficiency of In‐House Clear Aligners in Limited Orthodontic Treatment

**DOI:** 10.1111/ocr.70066

**Published:** 2025-11-24

**Authors:** Michael C. Kessler, Joon Han, George J. Eckert, Lana Helms, Jay A. Hughes, Phillip Wong, Carolina Frota, Vinicius Dutra, Hakan Turkkahraman, R. Scott Conley

**Affiliations:** ^1^ Department of Orthodontics and Oral Facial Genetics Indiana University School of Dentistry Indianapolis Indiana USA; ^2^ Indiana University School of Dentistry Indianapolis Indiana USA; ^3^ Department of Biostatistics and Health Data Science Indiana University School of Medicine Indianapolis Indiana USA; ^4^ Department of Oral Pathology, Medicine, and Radiology Indiana University School of Dentistry Indianapolis Indiana USA

**Keywords:** clear aligner appliances, orthodontic tooth movement, three‐dimensional imaging, treatment efficacy

## Abstract

**Objective:**

To evaluate the efficacy and efficiency of in‐house digital software and fabrication of clear aligners.

**Materials and Methods:**

This retrospective study analysed pre‐treatment, predicted and post‐treatment digital scans of 61 patients (42 females, 19 males) to assess the accuracy of predicted tooth movements. Planned and final scans were superimposed using best‐fit analysis in Geomagic Design X (Hexagon AB, Stockholm, Sweden). Distoincisal (DI), mesioincisal (MI) and gingival zenith (Z) landmarks were measured perpendicularly from the mid‐facial aspect, with differences < 0.5 mm deemed clinically acceptable. Data were analysed using repeated‐measures ANOVA with logarithmic transformation, and clinically acceptable movements were compared between groups using generalised estimating equations (GEE).

**Results:**

When evaluated individually, 91% of mandibular and 95% of maxillary landmarks showed clinically acceptable movement. When all three landmarks per tooth were below the threshold, 84% of mandibular and 88% of maxillary teeth met this criterion. At the case level, 48% of mandibular and 50% of maxillary cases achieved overall clinically acceptable movement. The greatest discrepancies were observed at the Z point on maxillary teeth (*p* < 0.05). Mandibular canine movements were more predictable than those of mandibular incisors (*p* < 0.05).

**Conclusion:**

In‐house aligner planning and fabrication demonstrated effective and efficient outcomes for limited treatment cases. Tooth movement was generally more predictable in the maxilla than in the mandible, with canines showing greater predictability than incisors. Torque movements in the maxilla exhibited the lowest accuracy.

## 1 Introduction

Clear aligner therapy (CAT) has experienced decades of material progression and software development in its utilisation as orthodontic treatment. From the on‐site production of tooth positioners [1] to digital setups with corporate manufacturing, technological advances have now increased practitioners' confidence in using clear aligners for the treatment of many malocclusions. In the initial years, neither the digital setup nor the outcome of corporate manufactured aligners was being studied to understand the success or failure of achieving planned tooth movements. Kravitz and colleagues found the mean accuracy of all tooth movements treated with Invisalign (Align Technology Inc., San Jose, CA, United States) to be around 41% [2]. A follow‐up study conducted by Kravitz and his team indicated one in every six patients required fixed traditional appliances (braces) following Invisalign with only 6% of patients finishing treatment without a refinement scan, and patients averaging 5 months longer than predicted [3]. While two studies do not provide sufficient evidence for the effectiveness of proprietary aligners, they established an accuracy baseline to monitor the progression of clear aligners from this company and many others. To date, there are approximately 30 marketed brands of aligners. Each of these has unique strengths and challenges meaning they must be evaluated as well. Among the more recent changes is that practitioners now possess the ability to fabricate in‐house aligners with three‐dimensional (3D) digital printers.

The digital age of dentistry and orthodontics started with the streamlining of electronic health records in the late 1990s and early 2000s [4]. Soon after, computers began to appear in every dental office and the use of physical impressions and gypsum casts decreased. The applications for digital workflow and 3D printing are expanding and unlocking the capabilities of this fairly new technology in orthodontics. Currently, practitioners are using in‐house printers for numerous applications including fabricating patient casts, surgical and temporomandibular dysfunction splints, removable retainers and clear aligners [5].

With the availability of 3D printing in office, the steady rise in costs for corporate clear aligners has led orthodontists to consider in‐house 3D printing to fabricate in‐office clear aligners. The advantages of in‐house fabrication include not only decreased costs but also a streamlined workflow with nearly complete autonomy from start to finish, leading to potential same‐day delivery of aligners to patients [6]. Previous studies have demonstrated that practitioners can use 3D printers to fabricate well‐fitting and comfortable aligners within a clinically acceptable error range [7].

While fabrication of in‐house aligners is possible, current research of their efficacy is still lacking. Sereewisai et al. performed a systematic review to evaluate digital setup versus the actual results with fixed appliances [8]. The results indicated that uLab digital setups (uLab Systems Inc., Memphis, Tennessee, United States) performed with a varying degree of accuracy but could be used for treatment plan presentations and visualisation of results.

With limited available evidence regarding the accuracy and treatment outcomes of in‐house CAT, it is imperative to test what is actually occurring as this treatment approach gains broader adoption. The aim of this study is to determine the efficacy and efficiency of in‐house digital software and fabrication of clear aligners.

## 2 Materials and Methods

### 2.1 Ethics

This retrospective study was granted exempt status by the Institutional Review Board (IRB) of Indiana University (Protocol #: 25348, Approved by 3 December 2024).

### 2.2 Power Analysis

A pre‐study sample size calculation was conducted. With a sample size of 60 cases, the two‐sided 95% confidence interval for the percentage of cases within 0.5 mm will have a maximum width of 27%. The comparisons will also be able to detect a 15% relative difference in the distance moved, based on a one‐sample *t*‐test calculation at 80% power and a two‐sided 5% significance level, assuming a standard deviation of 40%.

### 2.3 Study Sample

Digital models were obtained from intraoral scans collected at two separate private practice offices using the iTero Element Scanner (Align Technology Inc., San Jose, California). All scans were de‐identified and assigned random identification numbers prior to analysis. The two participating practitioners were selected for their comparable levels of clinical experience (> 25 years in practice) and extensive familiarity with clear aligner therapy (use of proprietary aligner systems for > 10 years and in‐house, orthodontist‐directed aligner setups for > 5 years). Both clinicians graduated from the same institution and currently serve as adjunct clinical professors at our institution, ensuring a consistent educational and clinical background. Although no formal calibration session was conducted, both practitioners routinely apply similar evidence‐based clinical workflows in their practices, which reduced procedural variability. While the inclusion of only two experienced clinicians may limit generalisability, this approach allowed for greater methodological consistency and control across all cases. Inclusion criteria were as follows: in‐house aligner fabrication with uLab software digital setup, pre‐treatment crowding or spacing less than 6 mm per arch, full dentition in the treated arch (excluding third molars), aligners changed every 7–10 days, no mid‐treatment corrections or changes, adequate digital scan margins and treatment time less than 12 months. Exclusion criteria included incomplete digital scans, < 3 mm of gingival margin and the presence of scan artefacts affecting visualisation or measurement. The final sample was comprised of 61 cases, 38 maxillary arches (28 females, 10 males) and 23 mandibular arches (14 females, 9 males).

### 2.4 Measurements

**FIGURE 1:**
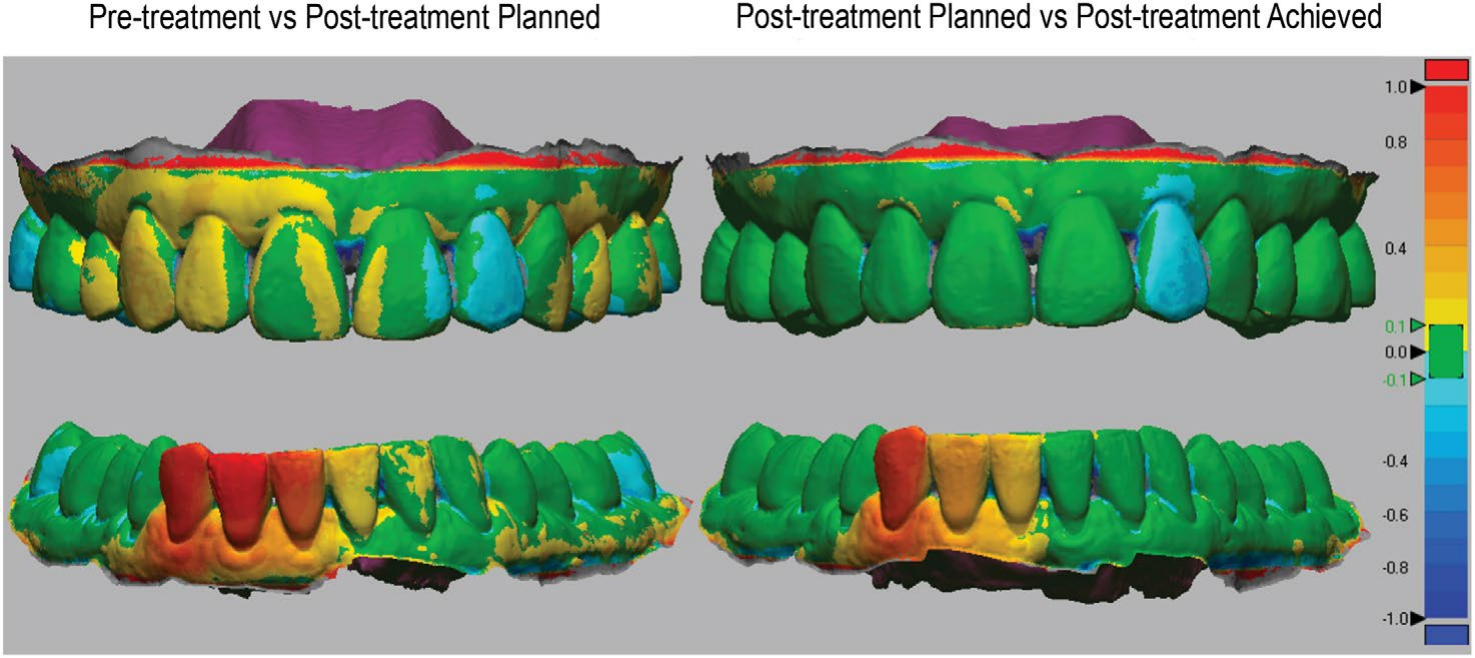
Images on the left side show the superimposition of the pre‐treatment with the planned post‐treatment models. Images on the right side show the superimposition of the planned with the actual post‐treatment models.

All measurements were performed by a single examiner (M.K.) who was blinded to both the planned and achieved outcomes to minimise observer bias and ensure measurement reliability. To perform the tooth movement analysis, paired models (pre‐treatment vs. post‐treatment and predicted post‐treatment vs. achieved post‐treatment) were superimposed using Geomagic Design X software (Hexagon AB, Stockholm, Sweden). Maxillary superimposition was completed by aligning the central fossae of the first molars and maxillary palatal rugae of the paired scans. Mandibular models were superimposed by aligning the central fossae of the first molars and the attached marginal soft tissue of the paired scans. The software then performed a best surface match technique to complete the superimposition (Figure 1).

To standardise the measurement technique, distoincisal (DI), mesioincisal (MI) and gingival zenith (Z) landmarks were selected for each tooth (Figure S1). The DI and MI points on the lateral incisors, central incisors and canines were identified as the intersection of the incisal line angle, mesio‐ or distofacial line angle and mesio‐ or distolingual line angle. The Z point for each tooth was identified as the most gingival position along the facial gingival margin. The points were identified while viewing perpendicular to the mid‐facial surface.

Movement accuracy was obtained by subtracting the total amount of tooth movement achieved from the planned tooth movement for each landmark. The distance between planned and achieved was categorised as clinically acceptable (< 0.5 mm) or not clinically acceptable (≥ 0.5 mm). The threshold for clinically acceptable movement was established from the American Board of Orthodontics (ABO) model grading system which notes that discrepancies < 0.5 mm receive no deductions indicating that differences smaller than this are not clinically relevant [9].

### 2.5 Statistical Analyses

Bland–Altman analysis and intraclass correlation coefficients (ICCs) were used to determine intra‐examiner repeatability of the measurements. The distance between planned and achieved tooth movements was calculated, summarised and analysed using repeated measures of variance analysis (RM‐ANOVA). A logarithmic transformation of the measurements was used in the ANOVAs. The percentage of clinically acceptable cases was summarised, using generalised estimate equation (GEE) methods for binary outcomes to compare between groups. A two‐sided 5% significance level was used for all tests. Analyses were performed using SAS version 9.4 (SAS Institute Inc., Cary, NC, US).

## 3 Results

### 3.1 Reliability Analyses

The results of the intra‐examiner repeatability analysis yielded an ICC of 0.92 indicating excellent reproducibility (Figure S2).

### 3.2 Descriptive Statistics

**FIGURE 2:**
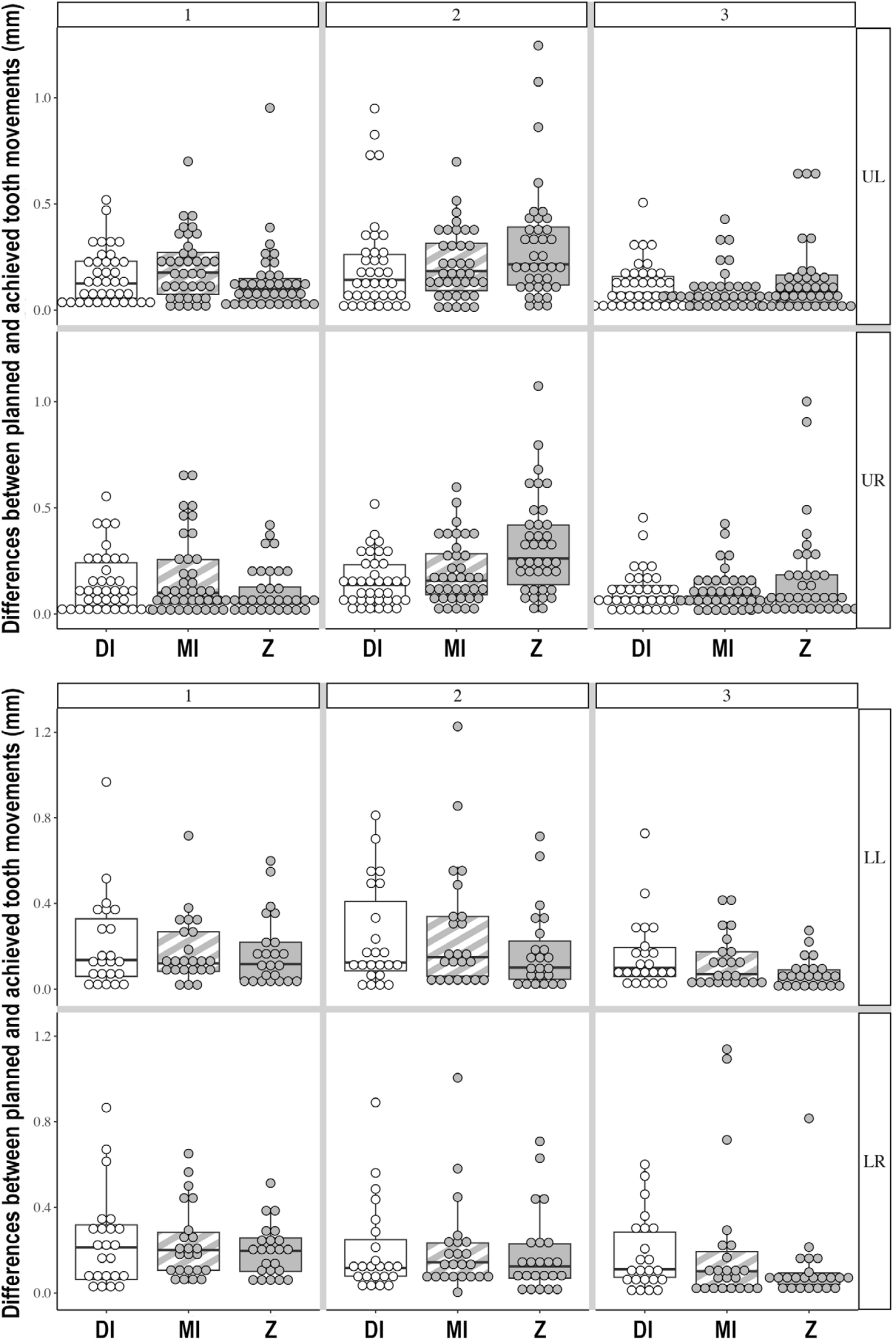
The boxplots showing the distribution of the differences between planned and achieved tooth movement.

The distribution of the differences between planned and achieved tooth movements across the teeth and points on teeth is shown with boxplots (Figure 2). The average planned tooth movement for the mandible was 0.28 mm with a range of 0–1.72 mm, while for the maxillary arch it was 0.24 mm with a range of 0–2.97 mm.

### 3.3 Efficacy Analyses

Descriptive statistics for the differences between planned and achieved movements for each tooth and landmark, as well as the percentage of cases within the clinically acceptable threshold of 0.5 mm, are presented in Table 1. The mean differences between planned and achieved movements for mandibular tooth landmarks ranged from 0.08 mm to 0.27 mm, while for maxillary teeth they ranged from 0.10 mm to 0.32 mm. When each landmark was evaluated individually, 91% of mandibular and 95% of maxillary landmarks demonstrated clinically acceptable movement. When a tooth was considered acceptable only if all three of its landmarks remained below the 0.5 mm threshold, 84% of mandibular teeth and 88% of maxillary teeth met this criterion. Finally, when all three landmarks of all teeth within a case were required to be below the threshold, 48% of mandibular cases and 50% of maxillary cases demonstrated overall clinically acceptable movement.

**TABLE 1:** Descriptive statistics for the differences between planned and achieved movements for each tooth and landmark, and the percentage of cases within the clinically acceptable threshold of 0.5 mm.

Tooth	Landmark	Mean	SD	Min	Max	% within 0.5 mm
LL1	DI	0.21	0.22	0.01	0.97	21/23 (91%)
	MI	0.18	0.16	0.01	0.72	22/23 (96%)
	Z	0.17	0.17	0.02	0.60	21/23 (91%)
LL2	DI	0.24	0.24	0.01	0.81	18/23 (78%)
	MI	0.27	0.30	0.03	1.23	19/23 (83%)
	Z	0.18	0.19	0.00	0.71	21/23 (91%)
LL3	DI	0.16	0.16	0.01	0.73	22/23 (96%)
	MI	0.13	0.12	0.01	0.42	23/23 (100%)
	Z	0.08	0.07	0.00	0.27	23/23 (100%)
LR1	DI	0.24	0.22	0.02	0.87	20/23 (87%)
	MI	0.23	0.17	0.04	0.65	20/23 (87%)
	Z	0.20	0.12	0.04	0.51	22/23 (96%)
LR2	DI	0.20	0.21	0.02	0.89	21/23 (91%)
	MI	0.21	0.22	0.00	1.01	21/23 (91%)
	Z	0.19	0.19	0.00	0.71	21/23 (91%)
LR3	DI	0.18	0.17	0.00	0.60	21/23 (91%)
	MI	0.21	0.32	0.01	1.14	20/23 (87%)
	Z	0.11	0.16	0.01	0.82	22/23 (96%)
UL1	DI	0.16	0.12	0.02	0.52	37/38 (97%)
	MI	0.20	0.15	0.00	0.70	37/38 (97%)
	Z	0.14	0.16	0.01	0.95	37/38 (97%)
UL2	DI	0.22	0.24	0.00	0.95	34/38 (89%)
	MI	0.21	0.16	0.00	0.70	36/38 (95%)
	Z	0.30	0.27	0.00	1.25	34/38 (89%)
UL3	DI	0.11	0.11	0.00	0.51	37/38 (97%)
	MI	0.10	0.09	0.00	0.43	38/38 (100%)
	Z	0.14	0.17	0.00	0.66	35/38 (92%)
UR1	DI	0.15	0.14	0.01	0.55	37/38 (97%)
	MI	0.18	0.19	0.00	0.66	35/38 (92%)
	Z	0.11	0.11	0.00	0.42	38/38 (100%)
UR2	DI	0.16	0.12	0.01	0.52	37/38 (97%)
	MI	0.20	0.15	0.01	0.60	36/38 (95%)
	Z	0.32	0.23	0.01	1.07	31/38 (82%)
UR3	DI	0.11	0.09	0.00	0.45	38/38 (100%)
	MI	0.12	0.10	0.00	0.42	38/38 (100%)
	Z	0.16	0.22	0.01	1.00	36/38 (95%)

The statistical comparisons of the differences between planned and achieved movement amounts are given in Table S1. In both jaws, tooth (e.g., central incisor, lateral incisor, canine etc.) had a significant main effect, suggesting that the measurement outcome differs significantly across teeth. However, the point on the teeth alone did not have a significant effect in either jaw. The interaction between tooth and point was not significant in the mandible, indicating the pattern of variation across teeth was similar at all points. However, the interaction between tooth and point on tooth was significant for the maxilla (*p* = 0.046) which means the relationship was not uniform and the variation across teeth changes depending on the point on the tooth. Given the significant interaction in the maxilla, further post hoc pairwise comparisons were performed first to investigate which specific maxillary teeth differ across points on tooth (Table 2). According to the results, the DI point was found to be less predictable for UL1 compared to UL3 and UR3, and for UR2 than UL3 (*p* < 0.05). All other tooth‐by‐tooth comparisons yielded no significant differences for the DI point (*p* > 0.05). As for the MI point, a similar result was found as UL1 was found to be less predictable than UL3 and UR3 (*p* < 0.05). Additionally, UL2 and UR2 both showed less predictability when compared to UL3 (*p* < 0.05). The most significant differences between planned and achieved movements were found for the Z point on the maxillary teeth, where both UR2 and UL2 were found to be significantly less predictable than the UR1, UL1, UL3 and UR3 (*p* < 0.05).

**TABLE 2:** Tooth by tooth comparisons of the differences between planned and achieved maxillary tooth movements for each landmark point.

Point	Tooth comparisons	Ratio of geometric means (95% CI)	*p*	*p* < 0.05
DI	UL1 & UL2	1.10 (0.65, 1.86)	0.708	
	UL1 > UL3	1.79 (1.16, 2.75)	0.009	*
	UL1 & UR1	1.34 (0.89, 2.02)	0.15	
	UL1 & UR2	1.05 (0.69, 1.60)	0.825	
	UL1 > UR3	1.62 (1.13, 2.33)	0.011	*
	UL2 & UL3	1.62 (0.89, 2.95)	0.108	
	UL2 & UR1	1.22 (0.65, 2.29)	0.527	
	UL2 & UR2	0.95 (0.56, 1.61)	0.846	
	UL2 & UR3	1.47 (0.86, 2.51)	0.151	
	UL3 & UR1	0.75 (0.45, 1.25)	0.259	
	UL3 < UR2	0.59 (0.35, 0.99)	0.045	*
	UL3 & UR3	0.91 (0.56, 1.47)	0.683	
	UR1 & UR2	0.78 (0.47, 1.29)	0.323	
	UR1 & UR3	1.21 (0.73, 1.99)	0.453	
	UR2 & UR3	1.55 (0.93, 2.57)	0.089	
MI	UL1 & UL2	1.01 (0.60, 1.71)	0.967	
	UL1 > UL3	2.09 (1.43, 3.03)	< 0.001	*
	UL1 & UR1	1.40 (0.90, 2.16)	0.132	
	UL1 & UR2	1.00 (0.64, 1.56)	0.989	
	UL1 > UR3	1.82 (1.06, 3.10)	0.03	*
	UL2 > UL3	2.06 (1.19, 3.59)	0.012	*
	UL2 & UR1	1.38 (0.79, 2.42)	0.254	
	UL2 & UR2	0.99 (0.58, 1.68)	0.958	
	UL2 & UR3	1.80 (0.99, 3.27)	0.055	
	UL3 & UR1	0.67 (0.39, 1.13)	0.131	
	UL3 < UR2	0.48 (0.29, 0.80)	0.006	*
	UL3 & UR3	0.87 (0.52, 1.45)	0.582	
	UR1 & UR2	0.71 (0.45, 1.13)	0.147	
	UR1 & UR3	1.30 (0.74, 2.30)	0.355	
	UR2 > UR3	1.82 (1.06, 3.14)	0.032	*
Z	UL1 < UL2	0.46 (0.29, 0.73)	0.002	*
	UL1 & UL3	1.37 (0.84, 2.24)	0.197	
	UL1 & UR1	1.50 (0.91, 2.46)	0.105	
	UL1 < UR2	0.39 (0.25, 0.59)	< 0.001	*
	UL1 & UR3	1.17 (0.78, 1.77)	0.431	
	UL2 > UL3	2.97 (1.74, 5.05)	< 0.001	*
	UL2 > UR1	3.24 (1.88, 5.58)	< 0.001	*
	UL2 & UR2	0.84 (0.52, 1.35)	0.459	
	UL2 > UR3	2.54 (1.52, 4.23)	0.001	*
	UL3 & UR1	1.09 (0.56, 2.11)	0.791	
	UL3 < UR2	0.28 (0.16, 0.51)	< 0.001	*
	UL3 & UR3	0.86 (0.56, 1.30)	0.457	
	UR1 < UR2	0.26 (0.16, 0.41)	< 0.001	*
	UR1 & UR3	0.78 (0.45, 1.37)	0.386	
	UR2 > UR3	3.03 (1.76, 5.21)	< 0.001	*

Abbreviations: CI, confidence interval; DI, disto‐incisal; MI, mesio‐incisal; Z, gingival zenith. * symbol denotes *p* < 0.05.

Second, which specific points on tooth differ across the maxillary teeth was investigated with pairwise comparisons (Table 3). The results showed that the MI point was found to be less predictable than the Z point on UL1 (*p* < 0.05). The Z point on UL2 was found to be less predictable than the DI point (*p* < 0.05). Similarly, the Z point on UR2 was found to be less predictable than both the DI and MI points. No significant differences were found between points on UL3, UR1 and UR3 (*p* > 0.05).

**TABLE 3:** Point by point comparisons of the differences between planned and achieved movement for each maxillary tooth.

Tooth	Point comparisons	Ratio of geometric means (95% CI)	*p*	*p* < 0.05
UL1	DI & MI	0.86 (0.55, 1.33)	0.48	
	DI & Z	1.29 (0.93, 1.78)	0.124	
	MI > Z	1.50 (1.01, 2.23)	0.043	*
UL2	DI & MI	0.79 (0.44, 1.39)	0.396	
	DI < Z	0.54 (0.33, 0.88)	0.015	*
	MI & Z	0.69 (0.39, 1.21)	0.185	
UL3	DI & MI	1.00 (0.55, 1.82)	0.994	
	DI & Z	0.99 (0.58, 1.67)	0.962	
	MI & Z	0.99 (0.58, 1.68)	0.969	
UR1	DI & MI	0.89 (0.59, 1.34)	0.563	
	DI & Z	1.44 (0.79, 2.62)	0.232	
	MI & Z	1.61 (0.87, 3.01)	0.127	
UR2	DI & MI	0.81 (0.53, 1.25)	0.336	
	DI < Z	0.48 (0.33, 0.68)	< 0.001	*
	MI < Z	0.59 (0.39, 0.88)	0.011	*
UR3	DI & MI	0.96 (0.63, 1.45)	0.837	
	DI & Z	0.93 (0.59, 1.46)	0.755	
	MI & Z	0.97 (0.56, 1.70)	0.921	

Abbreviations: CI, confidence interval; DI, disto‐incisal; MI, mesio‐incisal; Z, gingival zenith. * symbol denotes *p* < 0.05.

Finally, the differences between planned and achieved movements across the mandibular teeth were compared through pairwise comparisons (Table 4). According to the results, the movement of the mandibular canines was found to be more predictable than the mandibular central and lateral incisors (*p* < 0.05).

**TABLE 4:** Tooth by tooth comparison of the differences between planned and achieved movements for the mandibular teeth.

Tooth comparisons	Ratio of geometric means (95% CI)	*p*	*p* < 0.05
LL1 & LL2	0.91 (0.67, 1.24)	0.543	
LL1 > LL3	1.68 (1.12, 2.50)	0.014	*
LL1 < LR1	0.72 (0.55, 0.96)	0.026	*
LL1 & LR2	0.97 (0.67, 1.41)	0.881	
LL1 & LR3	1.48 (0.88, 2.50)	0.134	
LL2 > LL3	1.84 (1.38, 2.43)	< 0.001	*
LL2 & LR1	0.79 (0.55, 1.14)	0.195	
LL2 & LR2	1.07 (0.77, 1.48)	0.688	
LL2 & LR3	1.62 (0.96, 2.73)	0.068	
LL3 < LR1	0.43 (0.31, 0.61)	< 0.001	*
LL3 < LR2	0.58 (0.40, 0.84)	0.006	*
LL3 & LR3	0.88 (0.53, 1.47)	0.62	
LR1 & LR2	1.35 (0.93, 1.95)	0.109	
LR1 > LR3	2.05 (1.34, 3.13)	0.002	*
LR2 & LR3	1.52 (0.99, 2.34)	0.057	

Abbreviation: CI, confidence interval. * symbol denotes *p* < 0.05.

### 3.4 Efficiency and Cost Analyses

The results of the efficiency and cost analyses of the in‐house clear aligners are given in Table S2. The average treatment time for all patients was 6 ± 3.10 months. The maxillary arches averaged one tray more than the mandibular arches. The average cost to the doctor was about four times less than the cost incurred using proprietary aligners companies.

## 4 Discussion

Efficacy and efficiency are two key aspects of in‐house CAT that warrant evaluation to determine whether in‐house design and fabrication enhance clinical outcomes. In this study, efficacy refers to the ability of the digital treatment planning software to accurately predict the final dental outcome [10], while efficiency is defined as the estimated treatment time from the initial to the final tooth position. Although efficiency in orthodontics is sometimes expressed in terms of the number of aligners required to achieve a given movement [3, 11], our study focuses on treatment time as a more standardised and clinically relevant measure. This approach accounts for variations in aligner replacement intervals and refinement protocols, which can differ across systems and patients. All cases in this study followed a consistent refinement protocol, and treatment completion was defined by the achievement of the planned final tooth positions as verified through digital superimposition and clinical evaluation.

While several articles have reviewed clear aligner efficacy, they have generally focused on proprietary aligners rather than those fabricated by the orthodontist directly [12, 13]. To the best of the study team's knowledge, this is one of the few investigations evaluating the efficacy and efficiency of limited treatment in patients managed with in‐house, orthodontist‐fabricated clear aligners [14, 15]. However, it differs from previous studies in the following respects: first, this study focused on patients with mild to moderate tooth‐size/arch‐length discrepancies, whereas Sachdev et al. examined patients with minimal crowding (< 4 mm) and Jaber et al. [15] evaluated more complex cases (> 6 mm crowding). The variations in case severity among these studies highlight the need for future research encompassing the full spectrum of arch‐length deficiencies, from mild to severe. In addition, the present study analysed 61 patients, comprising 138 mandibular teeth and 228 maxillary teeth—substantially more than the sample in Sachdev et al. [14], which included 133 mandibular and 126 maxillary teeth.

The model superimposition technique used in the current study followed a best‐fit algorithm applied to relatively stable posterior regions, consistent with previous research. This approach has been employed in similar investigations, including Shahabuddin et al. [16] in their review of clear aligner effects on deep bite malocclusions, and Chan et al. [17] in their evaluation of clear aligner management of mandibular incisor extraction cases. The validity and repeatability of this superimposition method have been confirmed in studies such as Rahimi et al. [18], who used the same technique to track mandibular anterior tooth movement following two bonded retainer protocols, and Al‐Nadawi et al. [19], who assessed tooth movement efficacy under different aligner wear protocols. Furthermore, Adel et al. [20] and Awad et al. [21] have demonstrated that best‐fit surface registration using stable anatomical landmarks yields accurate and reproducible results for digital model superimposition, providing additional support for the reliability of the method applied in this study.

Within the current study, the maxillary lateral incisors exhibited significant differences between the planned and achieved torque given by the Z point. Combined, there is a notable pattern of less predictability of the maxillary lateral incisors compared to both canines and central incisors. This finding supports the results of the study by Castroflorio et al. [22] in that the inclination of the maxillary lateral incisors was one of the most challenging movements to control. It is known that torque placed on lateral incisors is a difficult movement as these teeth are thin, narrow, and do not provide seemingly sufficient crown structure and shape to support root movement through alveolar bone. The current study did not specifically study attachments, but Karsli et al. [23] noted that the placement of both facial and lingual attachments can aid in producing predicted torque.

Another key finding was that teeth or points on teeth that were planned for smaller movements tended to obtain the desired movement; that is, smaller movements were more accurately predicted. This was not observed by Sachdev et al. [14] who noted that as the predicted tooth movement increased so did the achieved tooth movement. A specific example of this from the current study was that the maxillary lateral incisors and the left central incisor were planned for larger movements yet exhibited significantly less predictability than the maxillary canines, which were planned for smaller movements. Interestingly the reduced accuracy observed in the lateral incisor with orthodontist‐fabricated clear aligners mirrors the reduced accuracy displayed by proprietary aligners [2]. Additionally, mandibular incisors that were planned for larger movements demonstrated significantly less accuracy than the mandibular left canine which was planned for smaller movement. This contrasts with the findings by Haouili et al. [24] that state that the canine often fails to achieve similar accuracy as other teeth with clear aligner treatment.

Rather than investigate the specific types of movement (labial, lingual, mesial, distal, intrusion, extrusion or rotation) that previous clear aligner studies have examined [2, 13, 14, 24, 25], the current study reviewed the difference between planned and achieved tooth movements from the perspective of individual points between teeth. Similar to the findings noted between teeth rather than points on teeth, the canines seemed to be more predictable with movements at the MI and DI points. However, no single tooth completely achieved 100% of the planned tooth movement implying that practitioners should include overcorrection into their planned tooth movements to achieve their desired clinical outcomes. This is supported by Palone et al. [26], who recommended overcorrection of the digital treatment plan to optimise the outcomes of clear aligner therapy. Castroflorio et al. [22] further support the need for overcorrection showing results that could be an outline to assist practitioners with an in‐depth review of which teeth need what type of overcorrection. On average, they found that most teeth need 30%–50% overcorrection to obtain predicted results.

In terms of cost‐effectiveness, this study found that the average cost to the practitioner for in‐house aligner fabrication was found to be around $150 for an average of six aligners. This finding was consistent with a recent study conducted by the American Association of Orthodontists (AAO) [27], which reported comparable cost estimates for in‐house aligner production. According to that study, the estimated costs for in‐house aligner fabrication included digital set‐up ($5.65 per file exported), 3D printing ($6.75 per model) and fabrication and packaging ($7.25 per aligner), resulting in a total of approximately $19.65 per aligner. For a minor tooth movement case requiring 14 aligners, the total cost was approximately $550. In contrast, the third‐party laboratory fee for an equivalent lite case was approximately $1349, yielding an estimated $799 cost savings per case when using in‐house production. However, these figures represent average estimates based on current market pricing and typical workflow costs. Fixed expenses such as scanner and printer acquisition, software licensing and staff training were not amortised in this analysis, as they vary substantially across practices and depend on treatment volume. A more comprehensive cost‐effectiveness analysis, incorporating these fixed and variable factors, is recommended for future research to better characterise the economic advantages of in‐house aligner systems.

## 5 Limitations and Future Directions for Research

This study has several limitations that should be acknowledged. First, the best‐fit algorithm used for superimposition relied primarily on the posterior dentition; however, molars may have experienced movement during treatment. The most probable change in the posterior segments would be intrusion resulting from aligner wear, which was not accounted for in the analysis. In addition, superimposition errors introduced by the software during initial alignment may have further reduced the accuracy of tooth movement measurements. This study also focused exclusively on the magnitude of tooth movement without evaluating its direction. Directional analysis is particularly important for assessing individual tooth movements, such as distinguishing between mesial and distal displacement or between labial and palatal torque. However, such analysis was beyond the scope of the present dataset and is recommended for future investigations. Another limitation of this study is the lack of data on the presence, type and placement of attachments, which may influence the accuracy of tooth movement [28, 29, 30], particularly torque prediction [31]. Future studies should incorporate this information to better assess its impact on model performance. Furthermore, this retrospective study lacked patient compliance data, such as self‐reported or objectively measured aligner wear time. Future prospective studies are warranted to incorporate this factor to better understand its influence on treatment outcomes. Finally, standardising the scanning software, printing technology and aligner materials across all cases would further enhance measurement accuracy and reduce variability.

## 6 Conclusions

Based on the findings of this study, in‐house planning and fabrication of clear aligners was found to be both efficacious and efficient, yielding clinically acceptable outcomes for cases involving limited treatment. Orthodontic tooth movement was generally more predictable in the maxilla than in the mandible. Among anterior teeth, canine movement exhibited greater predictability than that of the central and lateral incisors, particularly in the mandible. Torque movements in the maxilla demonstrated the lowest accuracy compared to other types of movement, such as contact point displacement.

## Author Contributions

Michael C. Kessler contributed to the conceptualisation, methodology, investigation and writing of the manuscript. Joon Han contributed to the data collection and measurements for the manuscript. George J. Eckert contributed to the methodology, formal statistical analysis and writing of the manuscript. Lana Helms contributed to the conceptualisation, methodology and provided samples needed for the research. Jay A. Hughes contributed to the conceptualisation, methodology and provided samples needed for the research. Phillip Wong contributed to the conceptualisation, methodology and investigation of the manuscript. Carolina Frota contributed to the conceptualisation, methodology, investigation and writing of the manuscript. Vinicius Dutra contributed to the conceptualisation, and methodology of the manuscript. Hakan Turkkahraman contributed to the conceptualisation, methodology, investigation, writing, revision, and supervision of the manuscript. R. Scott Conley contributed to the conceptualisation, methodology, investigation, writing, revision and supervision of the manuscript.

## Funding

The authors have nothing to report.

## Ethics Statement

All authors complied with all applicable research and patient care guidelines and regulations including HIPAA, IRB, institutional, state, federal, rules and regulations.

## Conflicts of Interest

The authors declare no conflicts of interest.

## Supporting information


**Figure S1:** Landmarks used in this study.
**Figure S2:** Bland–Altman plots for the intra‐examiner repeatability.
**Table S1:** The results of the repeated measures of variance analysis (RM‐ANOVA) for the differences between the predicted and achieved tooth movements.
**Table S2:** Efficiency and cost analyses of in‐house aligners.

## Data Availability

The data that supports the findings of this study are available in the Supporting Information of this article.
