# Supplementary material for: Efficacy and Efficiency of In‐House Clear Aligners in Limited Orthodontic Treatment
Source: Orthod Craniofac Res. 2025 Nov 24;29(1):186–95. doi: 10.1111/ocr.70066 (PMC12779182; doi:10.1111/ocr.70066)
Supplement: Supplementary file 1 — Figure S1: Landmarks used in this study. Figure S2: Bland–Altman plots for the intra‐examiner repeatability. Table S1: The results of the repeated measures of variance analysis (RM‐ANOVA) for the differences between the predicted and achieved tooth movements. Table S2: Efficiency and cost analyses of in‐house aligners. [file OCR-29-186-s001.docx]

**Supplemental Figure 1.** Landmarks used in this study.


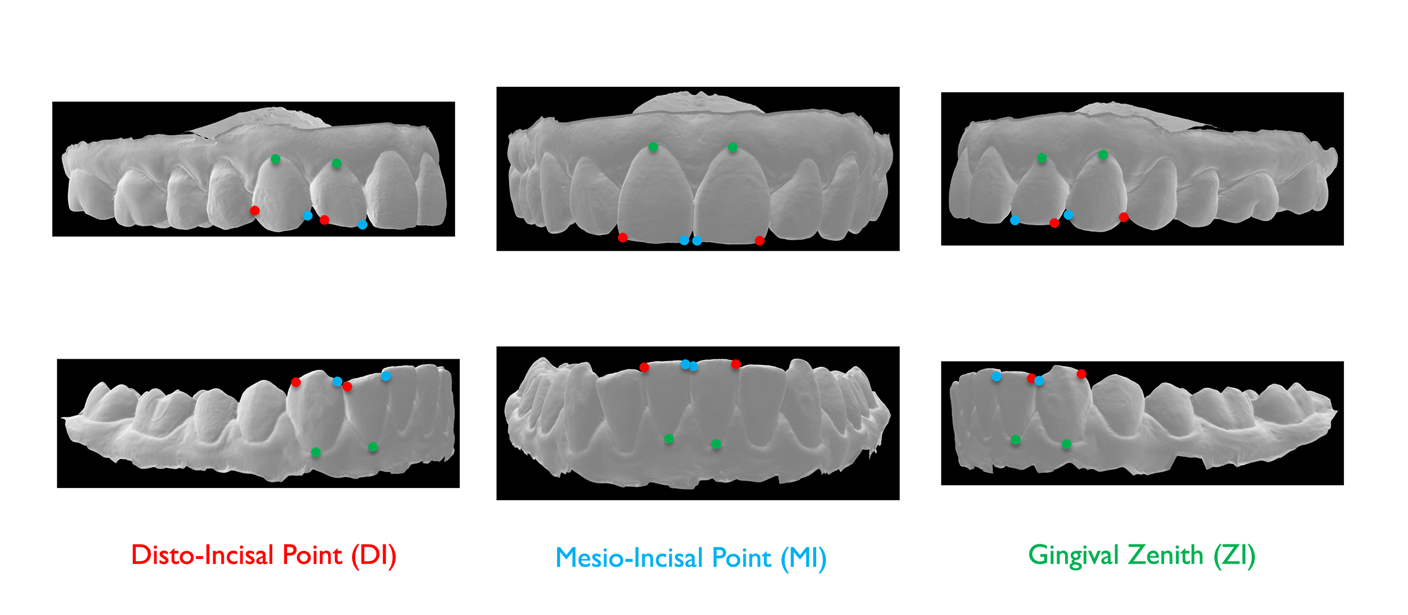


**Supplementary Figure 2.** Bland-Altman plots for the intra-examiner repeatability.


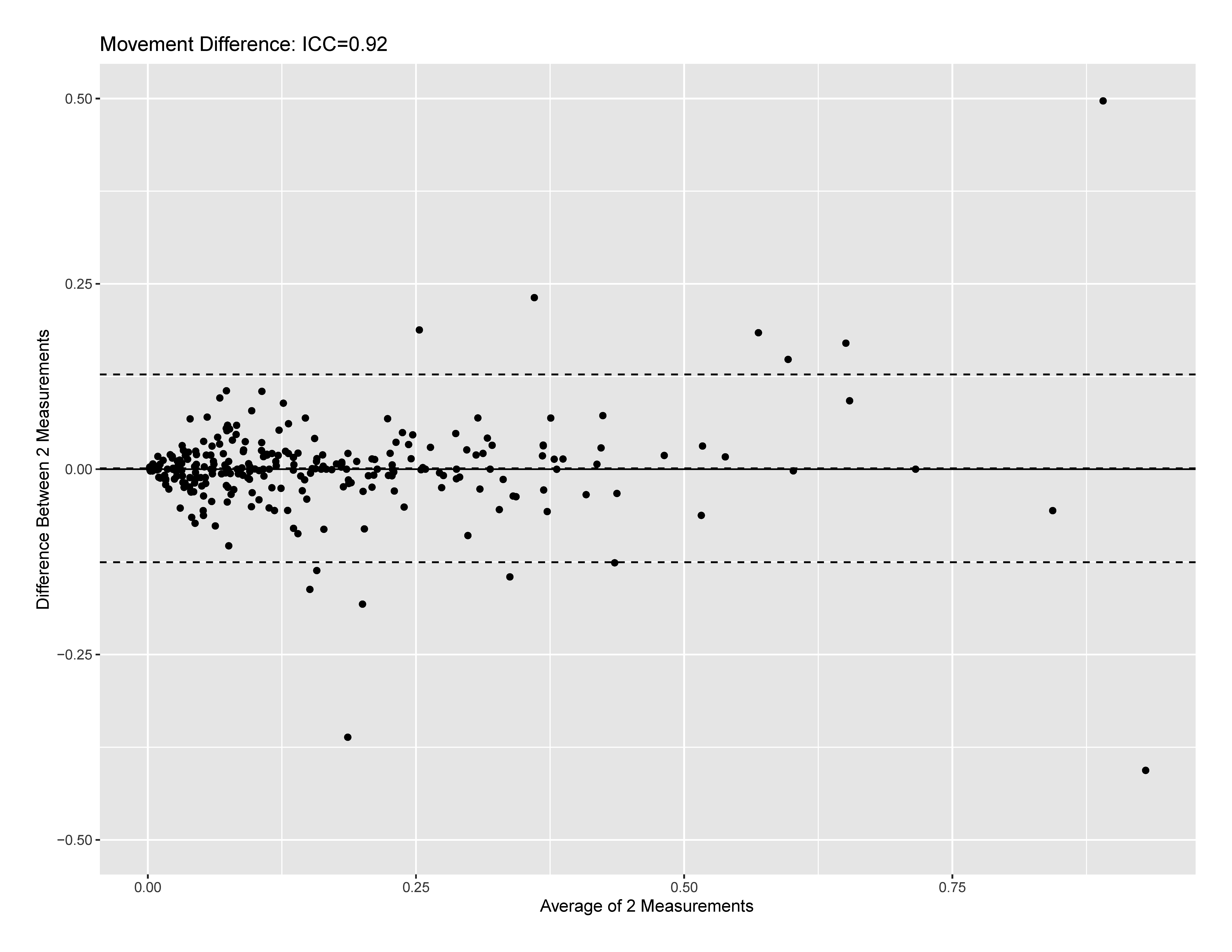


**Supplemental Table 1.** The results of the repeated measures of variance analysis (RM-ANOVA) for the differences between the predicted and achieved tooth movements.

| **Jaw** | **Effect** | **Num DF** | **Den DF** | **F-Value** | **P-value** | **P<.05?** |  |
| --- | --- | --- | --- | --- | --- | --- | --- |
| Mandible | tooth | 5 | 18 | 11.4 | <.001 | * |  |
| Mandible | point | 2 | 21 | 2.46 | 0.11 |  |  |
| Mandible | tooth*point | 10 | 13 | 0.5 | 0.86 |  |  |
| Maxilla | tooth | 5 | 33 | 8.29 | <.001 | * |  |
| Maxilla | point | 2 | 36 | 1.37 | 0.266 |  |  |
| Maxilla | tooth*point | 10 | 28 | 2.23 | 0.046 | * |  |

**Supplemental Table 2.** Efficiency and cost analyses of in-house aligners.

| **Treatment Time (months)** | Maxilla | 6.3 |
| --- | --- | --- |
|  | Mandible | 6.04 |
| **Number of Aligners** | Maxilla | 6.37 |
|  | Mandible | 5.04 |
| **Cost** | Doctor | $151.10 |
|  | Patient | $819.27 |
